# Ongoing mumps outbreak in Israel, January to August 2017

**DOI:** 10.2807/1560-7917.ES.2017.22.35.30605

**Published:** 2017-08-31

**Authors:** Viki Indenbaum, Judith M Hübschen, Chen Stein-Zamir, Ella Mendelson, Danit Sofer, Musa Hindiyeh, Emilia Anis, Nitza Abramson, Eric J Haas, Yochi Yosef, Larisa Dukhan, Shepherd Roee Singer

**Affiliations:** 1 National Center for Measles/Mumps/Rubella, Central Virology Laboratory, Ministry of Health, Sheba Medical Center, Tel Hashomer, Israel; 2 These authors contributed equally to this article and share first authorship; 3 Infectious Diseases Research Unit, Department of Infection and Immunity, Luxembourg Institute of Health, Esch-sur-Alzette, Luxembourg; 4 Jerusalem District Health Office, Ministry of Health, Jerusalem, Israel; 5 Division of Epidemiology, Ministry of Health, Jerusalem, Israel; 6 Southern District Health Office, Ministry of Health, Beersheba, Israel

**Keywords:** Mumps, outbreaks, surveillance, measles-mumps-rubella (MMR) vaccine, epidemiology, infection control

## Abstract

In Israel, 262 mumps cases were registered between 1 January and 28 August 2017 despite a vaccine coverage of ≥ 96%. The majority (56.5%) of cases were adolescents and young adults between 10 and 24 years of age. Nearly twice as many cases were reported in males than in females. Sequence information identified genotype G and suggested specific transmission chains in different religious communities, with the Muslim population in Jerusalem being most severely affected.

## Outbreak description

**Figure 1:**
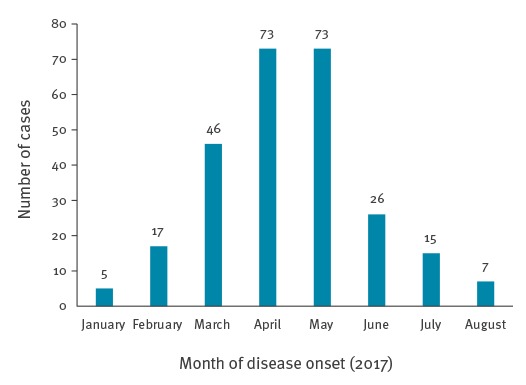
Mumps cases by month of disease onset, Israel, 1 January−28 August 2017 (n = 262)

Between 1 January and 28 August 2017, 262 cases of mumps were reported, most of them from Jerusalem (n = 190, 72.5%) and the Southern district (n = 40, 15.3%). Other health districts of Israel were more mildly affected: Central district (n = 14, 5.3%), Tel Aviv district (n = 8, 3.1%), Northern district (n = 6, 2.3%) and Haifa district (n = 4, 1.5%). Disease incidence peaked in April and May with 73 cases each (Figure 1). In addition to the seven cases reported in August, another 10 suspected cases (patients in whom a healthcare worker suspected mumps) are currently under investigation. Cases were defined as patients with acute onset of unilateral or bilateral tender, self-limited swelling of the parotid or other salivary gland, lasting two or more days and without other apparent cause [1].

**Figure 2:**
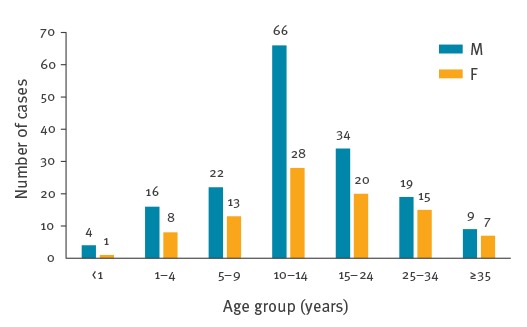
Mumps cases by age and sex, Israel, 1 January−28 August 2017 (n = 262)

Adolescents and young adults between 10 and 24 years of age were most affected (n = 148, 56.5%; median age: 13 years (range: < 1 to 69)). Cases were nearly twice as often reported among males than females (n = 170, 64.9% vs n = 92, 35.1%, Figure 2).

F: female; M: male.

All patients for whom the information was available were born in Israel (n = 260). The most affected ethnic groups were Arab Muslims (n = 183, 69.8%), Jews (n = 39, 14.9%) and Bedouin Muslims (n = 36, 13.7%). Vaccination status was determined for 53 patients, most of whom were vaccinated with either one (n = 20), or two doses (n = 27) of mumps-containing vaccine. Most of the known unvaccinated patients were either born before vaccine introduction (3/6) or were too young to be vaccinated (2/6). Disease complications were recorded in nine patients, mostly orchitis (8/9) and mainly in unvaccinated people (7/9). An 8-year old child with two documented doses of mumps-containing vaccine developed both meningitis and pancreatitis. A very high mumps IgG titre and no IgM antibodies were found in the samples collected from this case. Mumps virus was detected in the cerebrospinal fluid. No history of immune-suppression was elicited. No data were available about the number of patients hospitalised.

## Laboratory investigations

Samples for laboratory analysis were available from 79 patients (30.2%). Seventy-six cases were confirmed by detection of specific IgM antibodies (n = 48, Enzygnost Anti-Parotitis-Virus/IgM kit, Siemens, Germany) and/or PCR (n = 34, adapted from [2]). The complete mumps virus small hydrophobic (SH) gene was amplified for 20 patients using previously published primers [3].

**Figure 3:**
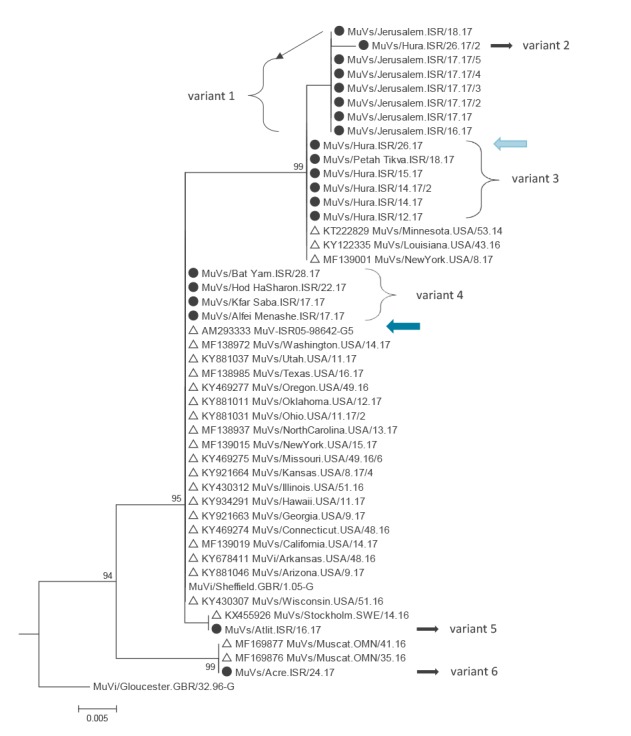
Part of a phylogenetic tree showing the mumps genotype G cluster in Israel, January to July 2017

Genotyping based on 316 nucleotides (nt) [4] using MEGA7 [5] showed that six different variants of genotype G were detected. Seven identical sequences were reported from a Muslim community in Jerusalem (variant 1, Figure 3) and one sequence differing by one nt found in a Bedouin child from a town near Beersheba (variant 2, Figure 3).

Based on 316 nt of the small hydrophobic (SH) gene and World Health Organization reference strains. The phylogenetic tree was generated by Molecular Evolutionary Genetic Analysis (MEGA) version 7.0.14 by neighbour-joining method with 1,000 bootstrap replicates; only bootstrap values of ≥ 70 are shown. Sequences from Israel (n = 20) are marked with a black dot, the closest BLAST fits are marked with triangles. The light blue arrow highlights the child with multiple complications, the dark blue arrow indicates a previous sequence reported from Israel.

The mumps sequences identified in this study were deposited in GenBank under accession numbers: MF788129−48*.

Neither variant 1 nor variant 2 have been reported to GenBank previously. A separate variant, previously also found in the United States (US), was detected in five patients from a Bedouin town near Beersheba and a Muslim child from a city near Tel Aviv (variant 3, Figure 3). No epidemiological link was found between the latter case and the cases in the south of the country, but the child had visited the West Bank region before developing mumps. One of the five Bedouin community patients was the previously mentioned child with complications (light blue arrow, Figure 3).

Four sequences identical to the World Health Organization Sheffield reference strain were found in patients from the Central and Tel Aviv health districts (variant 4, Figure 3). This sequence variant had been detected in 2005 in an army recruit from Israel (dark blue arrow, Figure 3) and was widely circulating in 2016−17 in the US. A single sequence differing by one nt from this variant was detected in a middle-aged Jewish adult from a village close to Haifa, who had attended a sports event in Jerusalem 2.5 weeks before symptom onset (variant 5, Figure 3). The only identical sequence found on GenBank originates from Sweden. Another single sequence differing by 7 nt from the Sheffield reference strain was found in a Druse child from the Northern district, who had visited France during the incubation period (variant 6, Figure 3). However, no recent mumps sequence information from France is available in GenBank. The only identical sequences found on GenBank were detected in 2016 in Oman.

## Control measures

In response to the increased number of mumps cases in 2017, control measures were undertaken according to the Israeli Ministry of Health (MoH) policy [6]. Catch-up immunisation campaigns were conducted in Jerusalem and a town near Beersheba among family and school contacts aged 1 to 17 years with a single dose of mumps-containing vaccine; for previously unvaccinated children, a second dose was recommended. Catch-up doses were offered free of charge. Contacts born before 1957, with a history of mumps illness or laboratory evidence of mumps immunity were considered immune. Medical institutions (hospitals, MoH and community clinics) were alerted in order to increase clinical awareness and sample collection from suspected cases.

## Discussion and conclusions

Mumps is a mandatory notifiable disease in Israel and cases are defined on the basis of the World Health Organization surveillance standard [1]. The last large outbreak of mumps occurred between 2009 and 2011, when more than 5,000 cases were reported [6].

Mumps vaccine was introduced into the routine childhood immunisation schedule in 1984 and vaccination with measles-mumps-rubella (MMR) vaccine was implemented in 1988. Currently two doses of mumps-containing vaccine are offered free of charge at 1 and 6 years of age. Vaccine coverage with the first dose has been at least 96% since 2006. Since 2013, coverage with the second dose has been consistently 95% and above and case counts declined to 47 in 2015 [7].

Despite high vaccination coverage, more than 260 cases of mumps were reported in Israel, mainly from the Jerusalem and Southern districts between 1 January and 28 August 2017. The Jerusalem area had been primarily affected by the previous outbreak in 2009 to 2011, when mostly ultra-orthodox Jews were affected [8,9]. In contrast, in the current outbreak, the majority of cases in Jerusalem (181/190, 95.3%) were reported in the Muslim community. In the Southern district, Bedouins were mostly affected (36/40) and in the Tel Aviv and Central districts, most cases were among Jews (20/22). Interestingly, the molecular data identified three chains of transmission (variant 1, 3 and 4) that largely correspond to these outbreaks, suggesting that mumps virus transmission is mostly restricted to members of the same ethnic group, with little spread between them. Variants 5 and 6 were distinct from the three outbreak variants which suggests independent introduction, although the source remains unknown. One case had visited France during the incubation period, but no recent mumps sequence information from France is available in GenBank to support a possible epidemiological link.

Many of the patient characteristics seen in 2017, such as male predominance, peak age in young adolescence and median age, largely overlap with what has been reported from the last large Jerusalem outbreak [9]. Several European countries with mumps outbreaks have also reported cases primarily in male adolescents and young adults, many of whom were vaccinated [10,11]. For the ongoing outbreak, information about vaccination status is so far available for only 53 patients, the majority of whom were vaccinated (n=47). While this is incomplete and preliminary information, previous findings raised questions about vaccine effectiveness, vaccination schedules, waning immunity, vaccine strains used, the potential need for an additional dose of MMR vaccine in outbreak situations, and characteristics of currently circulating mumps viruses [6,10-12].

In the ongoing mumps outbreak, the number of hospitalisations is unknown and complications were reported for only nine cases, most of them unvaccinated, supporting previous reports about less severe illness and/or lower rates of complications in previously vaccinated individuals [9,13-15].

Many residents of Israel travel abroad in the summer months, ca 933,000 in July this year alone [16]. In addition, the Hajj will take place in late August and last until early September, and major Jewish holidays are celebrated in September and October. For these reasons, there is an increased risk of mumps exportation and of further spread within the country. Thus, increased vigilance of healthcare workers and those working in public health is required.

In conclusion, mumps control remains a challenge in Israel in spite of very high vaccine coverage. Molecular data obtained during the ongoing outbreak proved to be valuable in helping to distinguish different transmission chains and independent virus importations. Research addressing the various issues related to long-term protection from currently circulating mumps strains is clearly warranted.

## Addendum

*The GenBank accession numbers were added on 1 September 2017.
