# Addendum for Euro Surveill. 2017;22(35)

**DOI:** 10.2807/1560-7917.ES.2017.22.36.30608

**Published:** 2017-09-07

**Authors:** Eurosurveillance editorial team

**Affiliations:** 1 European Centre for Disease Prevention and Control (ECDC), Stockholm, Sweden

In the article entitled ‘Ongoing mumps outbreak in Israel, January to August 2017’ by V Indenbaum et al., published on 31 August 2017, the GenBank accession numbers of the mumps virus sequences were added on 1 September 2017 at the request of the authors.

